# The relationship between medical student learning opportunities and preparedness for practice: a questionnaire study

**DOI:** 10.1186/1472-6920-14-223

**Published:** 2014-10-21

**Authors:** Bryan Burford, Victoria Whittle, Gillian HS Vance

**Affiliations:** School of Medical Education, Newcastle University, Ridley Building 1, Newcastle upon Tyne, NE1 7RU UK

**Keywords:** Preparedness, Simulation, Workplace learning, Transitions, Undergraduate medical education

## Abstract

**Background:**

Alongside providing a knowledge base and practical skills, undergraduate medical education must prepare graduates to immediately begin practice as qualified doctors. A significant challenge is to provide safe learning opportunities that will optimise students’ preparedness to start work. This study examined UK graduates’ preparedness for clinical practice, and their exposure to real-life and simulated immediate care scenarios during final year placements.

**Method:**

A questionnaire measuring students’ perceived preparedness, and their exposure to immediate care scenarios, was distributed to all new Foundation Year 1 doctors (F1s) attending an induction session in one region of the UK.

**Results:**

356 F1s responded to the questionnaire (91% response rate; 89% of cohort) and data from 344 graduates of UK medical schools were analysed. Respondents were generally prepared for practice, but many reported few ‘hands-on’ experiences of providing immediate care during final year placements (a median of 1–2 experiences).

Those who had 1–2 experiences reported no greater preparedness for acute management than those reporting no experience. Several exposures are necessary for a significant increase in perceived preparedness. Real-life experience was a better predictor of preparedness than simulated practice.

**Conclusions:**

Gaps still remain in medical students’ acute care experience, with a direct relationship to their perceived preparedness. The format and facilitation of placements may need to be addressed in order to enhance the quality of experience during final year.

**Electronic supplementary material:**

The online version of this article (doi:10.1186/1472-6920-14-223) contains supplementary material, which is available to authorized users.

## Background

The aim of medical education is to prepare students for practice, that is, to equip them with the skills and knowledge to enable them to begin work. ‘Preparedness’ also implies that they themselves are aware of their capabilities, and are confident in their ability to safely begin work.

For several years, however, there have been concerns that medical graduates in many countries are under-prepared for practice [1–5]. In the UK, recent work examining preparation for the first year of postgraduate training has identified particular concerns in areas of prescribing and of managing acutely unwell patients [6–8]. Medical students at graduation are expected by the regulator to be able to ‘provide immediate care in medical emergencies’ [9] (p. 22), while a more detailed Undergraduate Acute Care curriculum, made up of 88 relevant competencies, has been specified previously, and includes a requirement to ‘Demonstrate a systematic approach to the clinical assessment and timely management of the critically ill patient’ [10].

Preparedness has been linked to the amount of ‘hands-on’ practical experience gained before starting work [11, 12]. However, this experience may be limited for a number of reasons: patient safety, ‘competition’ for training opportunities with other grades or professions, or simply because of limited opportunities to see low frequency events. Enhanced undergraduate clinical experience has been shown to benefit perceived readiness and capability [13, 14], and to reduce stress around transition to F1 [15]. In some cases this experience may be included in a programme with simulated practice [16]. Other approaches using just simulated practice [17, 18] or classroom based training [5, 19] have also been attempted. The challenge remains, therefore, to identify the most appropriate means of supporting the transition of new graduates into the modern work-place.

In the light of these issues, the UK medical regulator, the General Medical Council (GMC), specified in its standards and outcomes for undergraduate medical education (*Tomorrow’s Doctors*) [9] that students gain practical experience in clinical teams and undertake most of the duties of Foundation Year 1 doctors in a ‘Student Assistantship’. In this, students are expected to "use practical and clinical skills", including "making recommendations for the prescription of drugs and managing acutely ill patients under the supervision of a qualified doctor" (p.55). Such placements are intended to provide students with a progressively more ‘central’ role in the team, while maintaining ‘legitimacy’ in the workplace [20].

This study considers students’ reported preparedness for different areas of practice since this change has been implemented, and how preparedness may be related to their placement training experiences during the final year.

Preparedness is operationalised as newly qualified doctors’ reported sense of being prepared for a number of areas of practice, with a questionnaire modelled on that used in an earlier study [21]. As such, the meaning of ‘preparedness’ is left to the respondents’ interpretation. While this may be problematic epistemologically [22], this operationalisation does allow some comparison with earlier studies.

### Research questions

Although precipitated by changes in policy in the UK, three primary research questions reflect international concerns in undergraduate medical education, namely how prepared doctors feel, and how that is related to the experience gained during clinical placements. The questions are:

(i) How prepared do newly qualified doctors feel for areas of practice specified in defined outcomes?
(ii) What experiences of acute care – real and simulated – are gained during final year placements?
(iii) How did reported preparedness for specified areas of practice vary with that experience?

## Method

### Questionnaire development

Questionnaire items were drawn from the outcomes specified in the current GMC standards set out in *Tomorrow’s Doctors* [9]. The majority were drawn from outcomes under the heading ‘The doctor as a practitioner’, rather than the other areas of ‘The doctor as a scholar and a scientist’ and ‘The doctor as a professional’. This was to ensure conciseness, but also reflect the primary interest in practical elements of care, which may have greatest implications for patient safety. All these items were responded to on a 5 point scale with anchors ‘Not at all prepared’ and ‘Fully prepared’.

Other items asked about specific experiences in final year placements, all specified in *Tomorrow’s Doctors*: if respondents had the opportunity to make recommendations for the prescription of drugs, had the opportunity to carry out common procedures on patients under supervision and had the opportunity to manage acutely unwell patients under supervision, with a five point scale (strongly disagree-strongly agree). The frequency of learning opportunities was addressed in the item ‘How many times have you had ‘hands-on’ experience of providing immediate care in a ‘real-life’ medical emergency?’. The definition of hands-on experience included a participatory role, ‘placing a cannula, airway manoeuvres etc.’, as well as a lead role, ‘assessing the patient, starting initial management, seeking help as appropriate’. This reflects the specific outcome in *Tomorrow’s Doctors* "Provide immediate care in medical emergencies" (Outcome 16) – indicating that graduates are expected to be able to perform this function, and that active participation is a realistic expectation of a final year placement [10]. To reflect the common use of simulation in undergraduate curricula, a further item asked ‘How many acute care simulation sessions did you attend in your final year? (e.g. sessions involving SimMan®)’.

Other items were also included but do not feature in the presentation of results here – the full questionnaire is available from the authors.

### Participants and procedure

Participants were new Foundation Year 1 doctors (F1s) starting work in one Foundation School in the UK. Questionnaires were distributed and completed in a lecture theatre at the local medical school where all new F1s were receiving the first session of their Foundation Programme induction, before dispersing to their employing trusts. Verbal instructions were given highlighting that the questionnaire was completely anonymous and optional, and that any or all questions could be omitted.

All questionnaires were completed during the induction session, with most returned immediately after the 20 minute time-slot available, although some were returned at the end of the session.

### Ethics statement

As the study was deemed evaluation and involved volunteer staff, ethical review was not required.

### Analysis

Statistical analysis was conducted using the R statistical programming language [23]. Where sub-group means are compared, non-parametric analyses are used due to non-normal distributions for some points. For multiple pairwise comparisons the Bonferroni correction for familywise error rate is applied to the interpretation of significant p-values. This means that where 10 comparisons are carried out, the 5% significance level is indicated by p < 0.005, and where 6 comparisons are carried out, it is indicated by p < 0.008.

Regression analysis on one preparedness item uses the clm() function for ordinal regression in R. The preparedness measure was treated as ordinal, while predictors were treated as continuous in order to retain their ordinal properties.

## Results

### Respondent profile

A total of 356 questionnaires were returned from 390 trainees (91.3% response rate, representing 89% of the entire F1 cohort). 213 respondents were graduates of the local medical school, 131 were graduates of other UK medical schools, and 9 were graduates from outside the UK. Three did not indicate where they had qualified. Although the preparedness of overseas graduates is an important concern, the small number of non-UK graduates does not allow any meaningful comparison to be made with the UK sample, and as they were from a number of different countries any summary data may be misleading. Consequently, the remainder of the analysis is based solely on the 344 confirmed UK graduates who will have had undergraduate experiences in line with GMC policy.

Of these, 134 indicated they were male (39%) and 210 female (61%). Table 1 summarise the frequencies of reported age groups. Distributions for sex and age are broadly in line with the population statistics for UK medical students [24].

**Table 1:** **Age groups of UK respondents**

Age group	Frequency (percent)
20-25	264 (76%)
26-30	70 (21%)
31-35	4 (1%)
36-40	1 (0.3%)
41 and over	4 (1%)

### Preparedness

The median response for all preparedness items was at the midpoint of the scale, with a skew towards the upper end of the scale indicating moderate preparedness among the population. However, a minority of individuals felt unprepared for each of the areas of practice. In particular, seven items were rated on the lower half of the scale by at least 10% of the sample (see Table 2. Distributions of all items are in Additional file 1). These low-rated items encompass a range of skills – clinical procedures, communication skills and prescribing – indicating it is not a particular class of skills or knowledge that is felt to be lacking, but specifics within all areas.

**Table 2:** **Frequencies of responses to preparedness items for which 10% or more of sample were unprepared**

Questionnaire items	1	2	3	4	5
Please indicate how prepared you are to begin foundation year 1 in the following areas….	Not at all prepared				Fully prepared
Prescribe dose and route of insulin, including use of sliding scales	12 (3.5%*)	75 (21.8%)	165 (48%)	76 (22.1%)	12 (3.5%)
	25.3%		48%	25.6%	
Prescribe, set up and monitor a blood transfusion	14 (4.1%)	51 (14.8%)	159 (46.2%)	105 (30.5%)	12 (3.5%)
	18.9%		46.2%	34%	
Wound care and basic wound dressing	10 (2.9%)	55 (16%)	118 (34.3%)	133 (38.7%)	23 (6.7%)
	18.9%		34.3%	45.4%	
Diagnose and manage acute medical emergencies.	2 (0.6%)	43 (12.5%)	144 (41.9%)	136 (39.5%)	15 (4.4%)
	13.1%		41.9%	43.9%	
Detect and report adverse drug reactions.	2 (0.6%)	40 (11.6%)	141 (41%)	130 (37.8%)	28 (8.1%)
	12.2%		41%	45.9%	
Carry out practical procedures: urinary catheterisation, skin suturing	4 (1.2%)	37 (10.8%)	133 (38.7%)	123 (35.8%)	42 (12.2%)
	12.0%		38.7%	48%	
Contribute to the care of patients and their families at the end of life	3 (0.9%)	31 (9%)	127 (36.9%)	148 (43%)	34 (9.9%)
	9.9%		36.9%	34%	

*NB. Percentages use the whole sample of 344 as denominator, not those who responded to the item, in order to provide an indication of prevalence with the sample as a whole.

### Experience

The questionnaire asked about different aspects of experience in the final year. Most respondents (321/344 = 93%) agreed they had experienced "at least one attachment where I assisted a junior doctor undertaking most of the duties of a Foundation Year 1 doctor" – the GMC’s definition of a Student Assistantship.

However, further questions asking about specific learning opportunities (relating to prescribing, practical procedures, and acute care management) identified gaps in learning. Table 3 summarises these responses. While nearly all respondents agreed that they had the opportunity to carry out practical procedures, large minorities disagreed or strongly disagreed that they had opportunities for prescribing or managing acutely unwell patients (23% and 30%, respectively). Final year placements may therefore be fulfilling their overall aims for many students, but a substantial number still feel they are not getting the experiences specified by national training policy.

**Table 3:** **Frequencies of responses to questions about experience in final year placements**

	1	2	3	4	5
	Strongly disagree				Strongly agree
I had the opportunity to make recommendations for the prescription of drugs (n = 342)	15 (4.4%)	64 (18.7%)	76 (22.2%)	115 (33.6%)	72 (21.1)
	23.1%		22.2%	54.7%	
I had the opportunity to carry out common procedures on patients under supervision (n = 343)	0	1 (0.3%)	24 (7.0%)	126 (36.7%)	192 (56.0%)
	0.3%		7%	92.7%	
I had the opportunity to manage acutely unwell patients under supervision (n = 341)	25 (7.3%)	76 (22.3%)	106 (31.1%)	92 (27.0%)	42 (12.3%)
	29.6%		31.1%	39.3%	

Two questions asked in more detail about the frequency of exposure to real-life and simulated immediate care scenarios (Table 4). The majority of the sample (n = 184; 53%) reported gaining fewer than three hands-on experiences in participatory as well as lead roles, with 42 (12%) not reporting any at all. Conversely, only 18% of respondents reported having had five or more experiences, despite completing a whole academic year of clinical placements.

**Table 4:** **Frequency (and percentage) of different amounts of experience in real life and simulated practice**

	None	1-2	3-4	5 or more
How many times have you had "hands on" experience of providing immediate care in a ‘real life’ medical emergency? (i.e. been a Participant or Lead in an acute care situation)* (n = 343)	42 (12%)	142 (41%)	97 (28%)	62 (18%)
How many acute care simulation sessions did you attend in your final Year? (i.e. sessions involving SimMan®) (n = 339)	8 (2%)	93 (27%)	144 (42%)	95 (28%)

*Lead defined as "assessed the patient, started initial management, sought help as appropriate"; Participant as "had a hands-on role e.g. placing a cannula, airway manoeuvres etc".

On the other hand, only eight respondents (2%) reported attending no acute care simulation sessions, while 239 (70%) reported three or more, suggesting that simulation has a substantial reach in undergraduate curricula. Most respondents reported receiving both real-life and simulated experience. Of the 42 who had no real-life experience, 40 indicated receiving only simulation. Of the eight who received no simulation, seven reported some real-life experience. One graduate responded ‘none’ to both questions.

### The relationship between experience and preparedness

To consider the relationship between experience and preparedness, a single item, drawn directly from the *Tomorrow’s Doctors* outcomes was selected as a key indicator of preparedness for acute practice, namely ‘How prepared do you feel to diagnose and manage acute medical emergencies?’. Responses to this item were considered against placement and simulation experience.

### Placement experience

**Figure 1:**
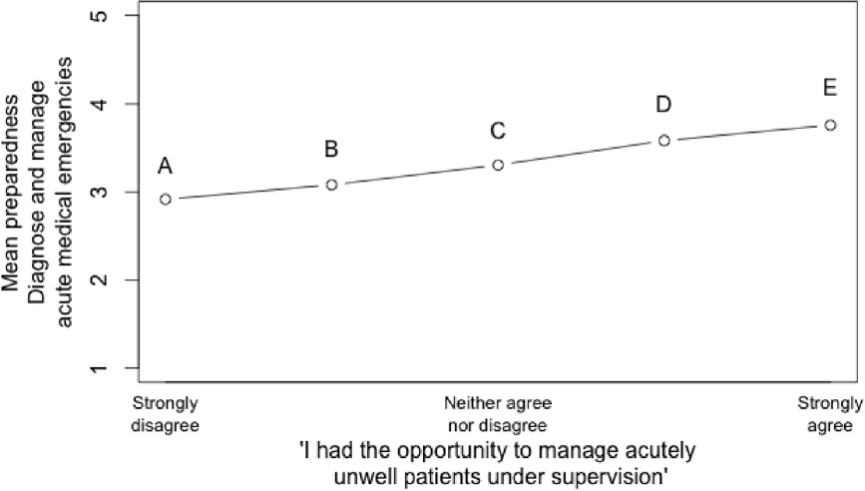
**Preparedness to manage acute patients plotted against perceived opportunity to manage acutely ill patients.**

Figure 1 shows how reported preparedness on this item increases with reported opportunity to manage acutely unwell patients. The difference between the levels of experience are significant by Kruskall-Wallis test (X = 38.1468, p < 0.001). This supports the intuitive expectation that greater (perceived) experience leads to greater preparedness.

However, pairwise comparisons between the points suggest that only the upper end of the experience scale is consistently associated with higher preparedness. Specifically, considering the labelled points in Figure 1, Mann Whitney U tests indicate only two points are significantly different from point A: point D (U = 605, p < 0.001) and point E (U = 219, p < 0.001). There are no differences between points A, B or C. Other significant differences are between the pairs B and D (U = 2276, p < 0.001), B and E (U = 804, p < 0.001) and C and E (U = 1399, p < 0.001).

**Figure 2:**
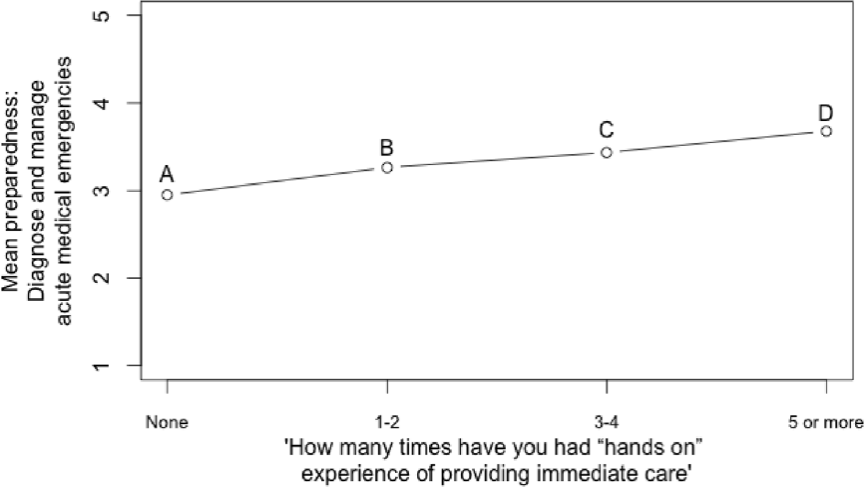
**Preparedness to manage acute patients plotted against number of experiences of providing immediate care.**

There is also a significant difference in preparedness when considered against the number of hands-on experiences of acute care (Kruskall-Wallis H = 25.4024, df = 3, p < 0.001, see Figure 2). However, pairwise comparison indicates that there is no significant difference between point A and point B, indicating that preparedness is no higher for those who have 1–2 experiences of immediate care than for those who have none. Point A is significantly different from point C (U = 1318, p < 0.001) and point D (U = 647, p < 0.001), while point B is also significantly different from point D (U = 2972, p < 0.001).

The implication is that at least three hands-on experiences are required for preparedness to be significantly greater than having no hands-on experiences. Reported preparedness is therefore associated with more experience, but this difference is only apparent with several exposures.

### Effect of simulation experience

**Figure 3:**
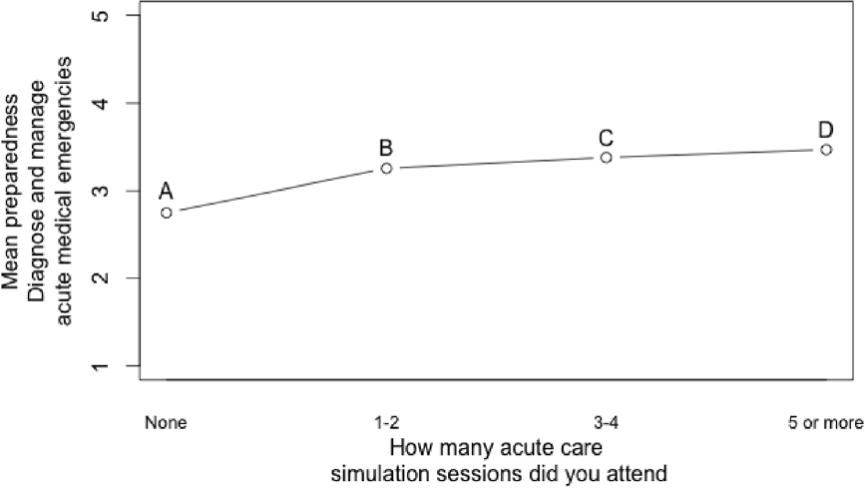
**Preparedness to manage acute patients plotted against number of simulation sessions attended.**

Experience of simulation also had a significant overall effect on preparedness (H = 9.9941, df = 3, p < 0.05), illustrated in Figure 3. However, the only significant pairwise comparison difference here is between point A (no experience) and point D (‘5 or more experiences’) (U = 177, p = 0.008). This indicates that experience of at least five simulation sessions is necessary for preparedness to be significantly higher than having had none.

### Real-life versus simulation?

The effects of real-life and simulated experience are confounded because most respondents had both types of experience. In order to estimate the relative impact of these, ordinal logistic regression analysis was carried out with real-life and simulated experience as predictors, and preparedness to diagnose and manage acute emergencies as the criterion. There is not a clear goodness of fit indicator (such as R^2^) for such an analysis. However, as we are interested in the relative importance of the two predictor variables, the explanatory power of the model as a whole is not a concern. Nonetheless, the interval_test() and scale_test() procedures in R [25] indicate that the proportional odds assumption is not violated and consequently that the model is appropriate.

The analysis indicated that real-life experience has a larger effect on preparedness than simulated experience does, although both are significant predictors of preparedness (for real-life experience, beta = 0.54, p < 0.0001; for simulation experience, beta = 0.26, p < 0.05). Therefore, while both types of experience influence preparedness, the influence of increased real-life experience is greater.

## Discussion

Newly qualified doctors completed a questionnaire which asked about their perceptions of their preparedness, and their final year experiences of real and simulated acute care. Reported preparedness was relatively high across most of 37 curriculum outcomes, although more than 10% of the sample felt unprepared for several items. These included practical, communication and prescribing-related skills.

Greater hands-on experience is associated with increased preparedness, supporting the implicit hypothesis which drives the introduction of practice-based placements – that the availability of more hands-on experience will improve preparedness. However, over half of the questionnaire respondents reported limited exposure to hands-on experience of immediate care (fewer than 3 occasions), and 12% reported having none at all (even with a relatively generous definition of participation in such cases). This suggests that the intended function of a Student Assistantship, to remedy the experiential gaps identified by Illing et al. [12, 26] and to rehearse students’ "eventual responsibilities as an F1 doctor [including] managing acutely ill patients" [9] (p55), has not been universal. While it seems that perceived preparedness overall is increasing [2, 21], concerns about junior doctors’ readiness to manage emergencies have been expressed for several decades [27], and it seems these latest efforts to provide experience may still not be providing the requisite preparation for practice.

As well as concerns about practice, and implicitly patient safety, arising from limited exposure, the literature shows that practical experience as an undergraduate may ameliorate stress [15]. The effects of the common lack of experience identified may therefore also be on the doctors’ own wellbeing as much as on their practice.

Literature on workplace learning identifies two routes of influence on individuals’ learning [28]. Firstly, there is the effect of the individual learner’s agency – their engagement with and pursuit of educational opportunities. This may be related to personality; preparedness has been found to vary with personality factors, as well as experience [11]. In relation to our findings, while it is intuitively appealing that there is a causal relationship between exposure and preparedness, and indeed that assumption has driven policy efforts to increase exposure, it is possible those who are more confident a priori are more likely to put themselves forward to gain such experiences. Attention to individual differences between students may be required as much as to the system of placements.

The second influence on experience is structural, represented in the ‘affordances’ of the workplace [28] – the circumstances of the specific workplace and working pattern which facilitate or prevent students being involved. These may be organisational or environmental, or arise from other members of staff. Some opportunities may be unavailable in some placements, for example ‘wound care and basic wound dressing’ will be more likely to arise in surgical than medical placements. (Although in a separate study we have found that such differences between learning opportunities in different placements are few [29]).

The initial introduction of the Assistantship intended to address these affordances, and to facilitate legitimate peripheral participation [20] rather than passive observation. We do not have baseline data for the frequency of learning opportunities before the Assistantship, but it seems that even if its introduction has facilitated positive change for many students, there remain many for whom it has not. Placements need to actively encourage and support participation [4, 30].

The question for educationalists is how to develop experience more effectively. Different solutions to these potential barriers are required. Where agency is the issue, development of students’ ability to recognise learning opportunities and have the assertiveness to take advantage of them may be necessary. Simulation may help here, through enhancing self-efficacy [31]. Simulation may also be the best way of gaining experience of low-frequency, high risk events, which may otherwise not be seen during student placements. However, our data indicate that while simulation has a role in increasing preparedness, real-life experience has a greater contribution.

Addressing other gaps may require more systematic changes to ensure that students gain experience in the appropriate range of specialties, and of necessary clinical conditions.

One way in which experience of the workplace may be enhanced is in ‘shadowing’, where a final year student follows the doctor whose post they will be taking on. While some implementations of the assistantship may allow a student to be attached to a single F1 for a period of time, matching students with their future F1 posts is rarely organisationally possible. The term is more usually used to refer to a period immediately before taking on the role, and this is the sense in which it is used by the GMC [9]. Such shadowing is valued and may increase preparedness [11, 32, 33], but does not eliminate unpreparedness [34]. The details of what is learnt or reinforced, and how, during shadowing remain under-studied.

### Limitations and strengths

The study was conducted in one location on a single day when trainees may be expected to be particularly anxious about starting work. This may be more true of those who were new to the area than those who had studied in the same place. On the other hand those who were local graduates may have been more aware of their recent student status in the same location, and the direction of any bias – if it exists – cannot be assumed. Analysis of our data (not reported in this paper for conciseness) found only slight differences between local and other UK graduates on two items.

The details of implementation of the Assistantship vary with individual hospitals. There is therefore variation within the region, as well as between regions. Transferability of findings cannot therefore be assumed, but rather should be considered in the light of particular local circumstances. However, the likely heterogeneity of experience of respondents suggests that findings should be taken seriously.

There are limitations on the interpretation of the findings here which arise from the use of anticipatory preparedness as the underlying construct. As we noted in the introduction, the use of ‘preparedness’ in this way was a pragmatic decision to allow comparison with an earlier study using a similar questionnaire [21], but we recognise the semantic and epistemological problems associated with its use [22]. Comparison with findings of other studies which operationalise preparedness as a *retrospective* judgement may therefore not be appropriate. When measured in advance of practice, as here, preparedness may be analogous to confidence, as a sense of ‘feeling prepared’, although this may be contaminated by respondents’ anxiety. However, while the accuracy of self-reported confidence can be assessed if there are appropriate measures of performance, the same is not true of reported preparedness, because there is not a clear definition or criterion of what it means to be prepared. Future work to examine the nature of ‘preparedness’, and more especially what the defining criteria are when translated into practice, is needed to develop educational strategies that will effectively bridge this gap.

## Conclusion

Practice placements for medical students as they approach qualification should provide opportunities for workplace learning. It is not enough for policy and curricula to state that such placements should take place, but rather attention needs to be paid to how undergraduate medical education can optimise learning in clinical workplaces, and improve legitimate peripheral participation. Simulated practice, while beneficial, does not replace experiences gained in real workplaces.

## Authors’ information

Bryan Burford is a Lecturer in Medical Education. He has a background in social and cognitive psychology, and has been working in medical education research since 2005.

Victoria Whittle is an MD candidate at Newcastle University. Her research interests are in the use of simulation as an educational strategy in acute care.

Gillian Vance is a Clinical Senior Lecturer (medical education) and Honorary paediatric consultant. Her interest in medical education arose through delivering clinical teaching and developing and managing courses. Accordingly, her research interests relate predominantly to student transition through the undergraduate medicine programme, and into the clinical workplace.

## Electronic supplementary material

Additional file 1: **Summary statistics and frequencies for all preparedness items.** (DOCX 19 KB)
