# Supplementary material for: The relationship between medical student learning opportunities and preparedness for practice: a questionnaire study
Source: BMC Med Educ. 2014 Oct 21;14:223. doi: 10.1186/1472-6920-14-223 (PMC4288662; doi:10.1186/1472-6920-14-223)
Supplement: Supplementary file 1 — Additional file 1: Summary statistics and frequencies for all preparedness items. (DOCX 19 KB) [file 12909_2014_1047_MOESM1_ESM.docx]

**Additional file 1: Summary statistics and frequencies for all preparedness items**

| ***Please indicate how prepared you are to begin Foundation Year 1 in the following areas*** | 1  Not at all prepared | 2 | 3 | 4 | 5  Fully prepared | Mean | SD |
| --- | --- | --- | --- | --- | --- | --- | --- |
| 1. Take and record a patient's medical history, including family and social history | 0 | 1 | 5 | 175 | 163 | 4.45 | 0.54 |
| 2. Elicit patients’ questions, their understanding of their condition and treatment options, and their views, concerns, values and preferences | 0 | 1 | 11 | 145 | 187 | 4.51 | 0.58 |
| 3. Perform a full physical examination | 0 | 2 | 36 | 213 | 93 | 4.15 | 0.61 |
| 4. Perform a mental-state examination | 2 | 25 | 101 | 172 | 44 | 3.67 | 0.81 |
| 5. Make an initial assessment of a patient's problems and formulate a differential diagnosis | 0 | 6 | 98 | 197 | 42 | 3.80 | 0.66 |
| 6. Formulate a plan for investigation and interpret the result | 0 | 12 | 132 | 172 | 27 | 3.62 | 0.68 |
| 7. Formulate a plan for treatment, management and discharge | 1 | 32 | 179 | 122 | 8 | 3.30 | 0.68 |
| 8. Make clinical judgements and decisions, based on the available evidence, in conjunction with colleagues | 0 | 19 | 122 | 175 | 26 | 3.61 | 0.71 |
| 9. Contribute to the care of patients and their families at the end of life | 3 | 31 | 127 | 148 | 34 | 3.52 | 0.83 |
| 10. Communicate effectively with colleagues from a variety of professions | 0 | 4 | 37 | 181 | 120 | 4.22 | 0.68 |
| 11. Communicate clearly, sensitively and effectively with patients, relatives or other carers | 0 | 3 | 29 | 167 | 145 | 4.32 | 0.66 |
| 12. Communicate appropriately in difficult circumstances (e.g. with difficult or violent patients, when breaking bad news, or with vulnerable patients) | 2 | 25 | 128 | 141 | 47 | 3.60 | 0.83 |
| 13. Assess and recognise the severity of a clinical presentation and a need for immediate emergency care | 1 | 24 | 106 | 170 | 38 | 3.65 | 0.78 |
| 14. Diagnose and manage acute medical emergencies. | 2 | 43 | 144 | 136 | 15 | 3.35 | 0.78 |
| 15. Provide cardio-pulmonary resuscitation or direct other team members to carry out resuscitation | 3 | 27 | 78 | 168 | 61 | 3.76 | 0.87 |
| 16. Plan appropriate drug therapy for common indications, including pain and distress | 2 | 18 | 120 | 174 | 26 | 3.60 | 0.73 |
| 17. Provide a safe and legal prescription | 1 | 13 | 85 | 175 | 69 | 3.87 | 0.78 |
| 18. Calculate appropriate drug doses | 3 | 21 | 97 | 165 | 58 | 3.74 | 0.84 |
| 19. Detect and report adverse drug reactions. | 2 | 40 | 141 | 130 | 28 | 3.42 | 0.82 |
| 20. Keep accurate, legible and complete clinical records | 0 | 3 | 40 | 178 | 121 | 4.22 | 0.68 |
| 21. Carry out baseline observations: measuring body temperature, pulse rate, blood pressure, transcutaneous oxygen monitoring (saturation monitoring) | 0 | 2 | 5 | 70 | 266 | 4.75 | 0.50 |
| 22. Carry out practical procedures: venepuncture, taking blood cultures, measuring blood glucose | 0 | 4 | 21 | 156 | 162 | 4.39 | 0.66 |
| 23. Establish peripheral intravenous access (cannulation) | 0 | 7 | 53 | 156 | 128 | 4.18 | 0.76 |
| 24. Perform and interpret a 12-lead electrocardiograph | 1 | 10 | 75 | 147 | 109 | 4.03 | 0.83 |
| 25. Carry out basic respiratory function tests | 0 | 16 | 65 | 179 | 81 | 3.95 | 0.78 |
| 26. Carry out practical procedures: urine multi dipstick test, taking nose, throat and skin swabs, pregnancy test | 0 | 10 | 46 | 152 | 135 | 4.20 | 0.78 |
| 27. Administer oxygen | 0 | 9 | 29 | 156 | 148 | 4.30 | 0.73 |
| 28. Prescribe dose and route of insulin, including use of sliding scales | 12 | 75 | 165 | 76 | 12 | 3.00 | 0.85 |
| 29. Administer subcutaneous and intramuscular injections | 1 | 20 | 81 | 152 | 89 | 3.90 | 0.86 |
| 30. Carry out practical procedures: urinary catheterisation, skin suturing | 4 | 37 | 133 | 123 | 42 | 3.48 | 0.89 |
| 31. Wound care and basic wound dressing | 10 | 55 | 118 | 133 | 23 | 3.31 | 0.92 |
| 32. Infection control (e.g. hand washing, use of personal protective equipment, safe disposal of waste and sharps) | 0 | 2 | 5 | 82 | 254 | 4.71 | 0.52 |
| 33. Prescribe fluids for intravenous infusion and set up infusion device | 5 | 26 | 102 | 168 | 38 | 3.61 | 0.84 |
| 34. Prescribe, set up and monitor a blood transfusion | 14 | 51 | 159 | 105 | 12 | 3.15 | 0.86 |
| 35. Hand over care of a patient | 0 | 13 | 92 | 187 | 50 | 3.80 | 0.73 |
| 36. Know when to seek help from a senior colleague | 0 | 8 | 36 | 205 | 92 | 4.12 | 0.68 |
| 37. Learn and work effectively within a multi-professional team | 0 | 4 | 23 | 177 | 139 | 4.31 | 0.65 |
